# Investigating the impact of a motion capture system on Microsoft Kinect v2 recordings: A caution for using the technologies together

**DOI:** 10.1371/journal.pone.0204052

**Published:** 2018-09-14

**Authors:** MReza Naeemabadi, Birthe Dinesen, Ole Kæseler Andersen, John Hansen

**Affiliations:** 1 Laboratory of Welfare Technologies—Telehealth and Telerehabilitation, Integrative Neuroscience Research Group, SMI, Department of Health Science and Technology, Faculty of Medicine, Aalborg University, Aalborg, Denmark; 2 Integrative Neuroscience Research Group, SMI, Department of Health Science and Technology, Faculty of Medicine, Aalborg University, Aalborg, Denmark; 3 Laboratory for Cardio-Technology, Medical Informatics Group, Department of Health Science and Technology, Faculty of Medicine, Aalborg University, Aalborg, Denmark; 4 Laboratory of Welfare Technologies—Telehealth and Telerehabilitation, SMI, Department of Health Science and Technology, Faculty of Medicine, Aalborg University, Aalborg, Denmark; Tokai University, JAPAN

## Abstract

Microsoft Kinect sensors are considered to be low-cost popular RGB-D sensors and are widely employed in various applications. Consequently, several studies have been conducted to evaluate the reliability and validity of Microsoft Kinect sensors, and noise models have been proposed for the sensors. Several studies utilized motion capture systems as a golden standard to assess the Microsoft Kinect sensors, and none of them reported interference between Kinect sensors and motion capture systems. This study aimed to investigate possible interference between a golden standard (i.e., Qualisys) and Microsoft Kinect v2. The depth recordings of Microsoft Kinect sensors were processed to estimate the intensity of interference. A flat non-reflective surface was utilized, and smoothness of the surface was measured using Microsoft Kinect v2 in absence and presence of an active motion capture system. The recording was repeated in five different distances. The results indicated that Microsoft Kinect v2 is distorted by the motion capture system and the distortion is increasing by increasing distance between Kinect and region of interest. Regarding the results, it can be concluded that the golden standard motion capture system is robust against interference from the Microsoft Kinect sensors.

## Introduction

In 2010, Microsoft, in cooperation with Prime Sense, introduced an RGB-D camera called “Kinect”. Initially, the Microsoft Kinect was developed as a gesture-based game controller for Microsoft Xbox 360. This device is equipped with RGB and near-infrared (NIR) sensors and NIR projector. It represents the depth information of viewing areas based on a structured light principle [1–3]. Within a year, Microsoft released official drivers and a software development kit (SDK) for Kinect for non-commercial use [1]. Microsoft Kinect SDK v1.8 [4] Open Kinect SDK [5], and OpenNI SDK [6] were developed to generate human joint skeleton in 3-dimensional space based on the captured information. Hence, the Kinect sensor is not only utilized as an RGB-D sensor but also as a natural user interface and frequently employed as a marker-less human motion tracking system in robotic applications [7,8], posture and daily activities [9–11], rehabilitation [12–14], virtual reality, and exergames [14–16].

Four years later, Microsoft improved the Kinect sensor capabilities and released the second generation of the Microsoft Kinect. The sensor’s specifications in this generation were significantly enhanced and embedded with a Full-HD RGB camera; the main improvement could be summarized regarding advances in generating a depth map. The second generation of Kinect, called “Kinect Xbox One” or “Kinect v2” in the literature generates depth information of the scanned area based on time-of-flight (ToF) principle [17,18].

The Microsoft Kinect sensors SDKs represent an estimation of body joints in head, torso, upper and lower limbs in 3-dimensional space. Hence, many scientific studies have been conducted to evaluate the reliability and validity of the calculated skeleton joints [11,19–24]. The majority of these studies employed marker-based motion capture systems as a golden standard. In general, these motion capture systems are equipped with several IR cameras with a built-in array of infrared LEDs illuminating retroreflective markers placed at strategic positions on the body. The cameras usually surround the area of interest. The tracking software collects data from all the cameras and estimates position of retroreflective markers based on triangulation. Therefore, IR retroreflective markers are mounted on the moving object of interest, and according to the marker position, the trajectory of the object moving in space is estimated.

Since all these systems are equipped with IR spectrum cameras and capture infrared images, interference between the Kinect sensors and motion capture systems is possible, which might result an added noise in the recordings.

Previously, several studies found an apparent interference between two Kinect sensors in the same generation; none of them evaluated or discussed interference between Kinect sensors and golden standard motion capture systems [9,19,23–30].

A wide variety of skeleton and posture tracking algorithms are proposed for the Microsoft Kinect sensors such as Microsoft Kinect skeleton SDK, Open NI SDK, and several other custom algorithms [31–34]. The skeleton tracking algorithms for Microsoft Kinect sensors are employing the estimated depth maps as raw input data to generate the 3D skeleton output. These algorithms calculate the position of the predefined joints in 3D space by extracting body point clouds and segmenting body parts from the depth maps. Thus, distortion in estimated depth maps might lead to inaccuracies in the generated 3D skeleton. Therefore, in this study, distortion in the acquired depth images were the focus of our inquiry.

In this study, it was hypothesized that the motion capture system will not interfere with Microsoft Kinect v2 sensor depth recordings. Consequently, the primary aim of this study was to investigate the impact of passive and active interference (introduced by retroreflective markers and cameras) on the depth recordings of Microsoft Kinect v2.

## Principle behind devices

### Microsoft Kinect sensors

**Fig 1:**
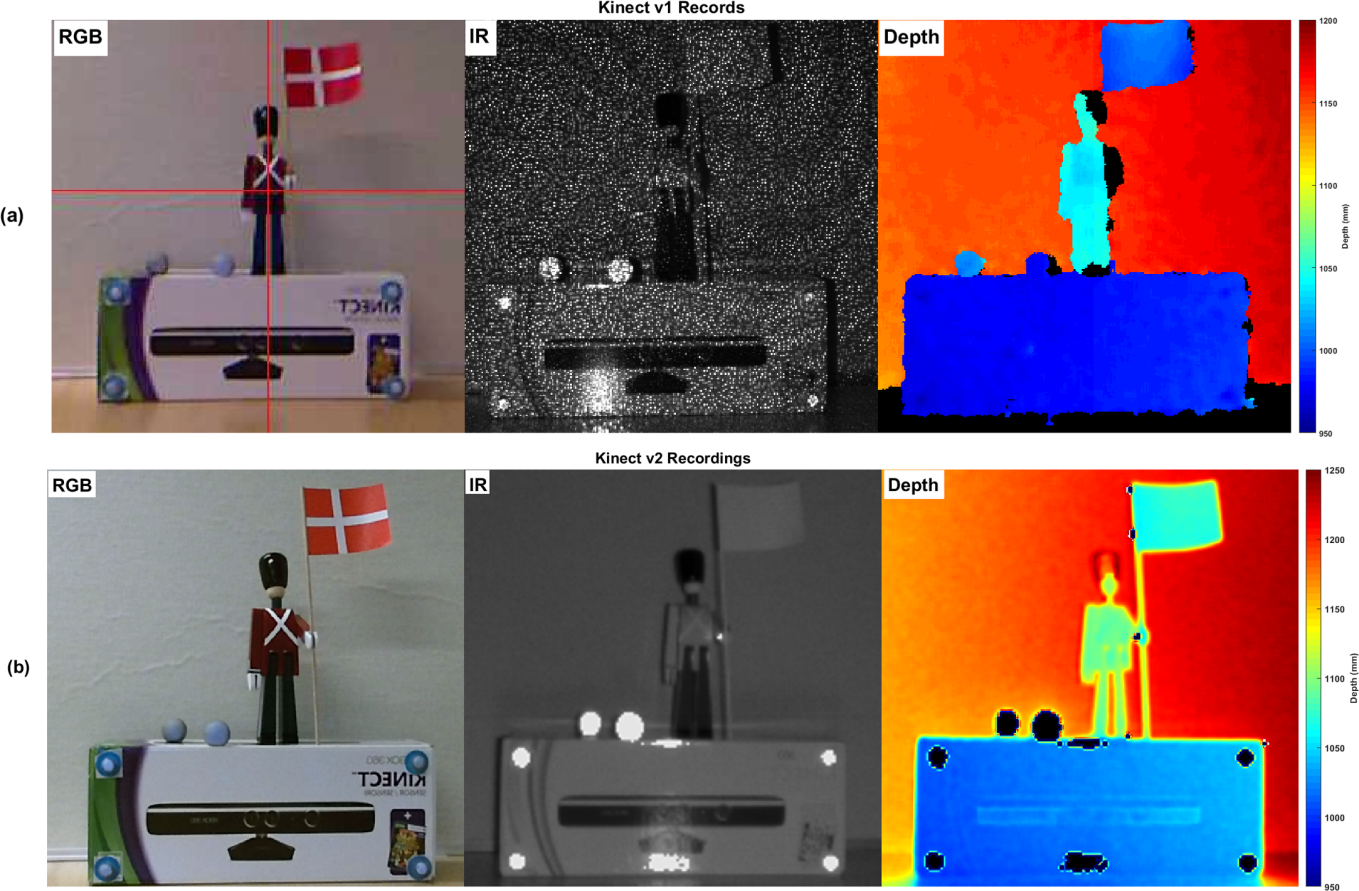
Microsoft Kinect records. (a) RGB and infrared records of Kinect v1 and corresponding depth map. The black pixels in the depth image reflect areas of unknown depth, which in this record is due to the out-of-record boundary, noise, and shadow effect on the left side. (b) RGB and IR images of Kinect v2 sensor and the corresponding estimated depth information (right panel), the black pixels in the depth image reflect areas of unknown depth.

The first generation of Microsoft Kinect, which in this study is called “Kinect v1”, emits a speckle pattern using infrared laser projectors. Kinect v1 estimates the depth map using a structured-light method. In this approach, the disparity of reflected speckle pattern in the captured image is compared with a reflected speckle pattern at the known distance. More details about Kinect v1 are available in [35–38]. Fig 1A illustrates an infrared record with the corresponding generated depth map of the seen RGB sight.

The second generation of Microsoft Kinect, which in this paper is called “Kinect v2”, is equipped with higher resolution image sensors and wider horizontal and vertical field of view (FOV). The operational range and resolution of Kinect v2 are enhanced by using a time-of-flight technique to estimate the depth (see Fig 1B).

The Kinect v2 laser projector emits square waveform NIR lights with known frequencies and receives the reflected lights from the object using a CMOS sensor with the very high sampling rate (2 GS/s). Apparently, by increasing the modulation frequency, uncertainty in depth is decreased, but this occurs at the cost of increased aliasing. Microsoft Kinect v2 employs two high modulation frequencies of 80 MHz and 120 MHz and a low modulation frequency of 16MHz to eliminate this ambiguity while acquiring less uncertainty in depth. In addition to the three-intensity modulations, phase reconstructions with 0˚, 120˚, and 240˚ phase shift are utilized to construct depth information. Further information about Kinect v2 and ToF cameras are available in [18,39,40].

### Marker-based motion capture systems

Motion capture systems are used for tracking fast human activities in 3-dimensions precisely, and the main principle of these systems is considered as the golden standard in motion tracking when assessing Kinect system performance. The marker-based motion capture systems (MB-MoCap) are generally divided into two categories: active marker and passive marker-based systems. However, both categories utilize a triangulation method to estimate the position of the markers in the covered space. The active markers emit light, while the passive markers reflect the emitted light from arrays of LEDs that are mounted on the cameras. A passive marker is often called a “retroreflective marker”. It should be mentioned that the majority of passive marker motion capture systems are also compatible with the active markers. Table 1 compares the most common marker-based motion capture systems.

**Table 1:** Comparison of the most common motion capture system.

Model	Marker Type	Working wavelength
Vicon MX (Vicon Motion Systems, Oxford, United Kingdom)	Passive	620, 780, 850nm
Qualisys Oqus (Qualisys AB, Gothenburg, Sweden)	Passive	850nm
Optotrak Certus (Northern Digital Inc, Ontario, Canada)	Active	900nm
PhaseSpace (Phoenix Technologies Inc, California, US)	Active	Red, NIR
PTI VZ4000 (Phoenix Technologies Inc, California, US)	Active	N.A.
Raptor Motion Analysis (Motion Analysis Corporation, Santa Rosa, USA)	Passive	750nm

### Emitted lights spectrum

**Fig 2:**
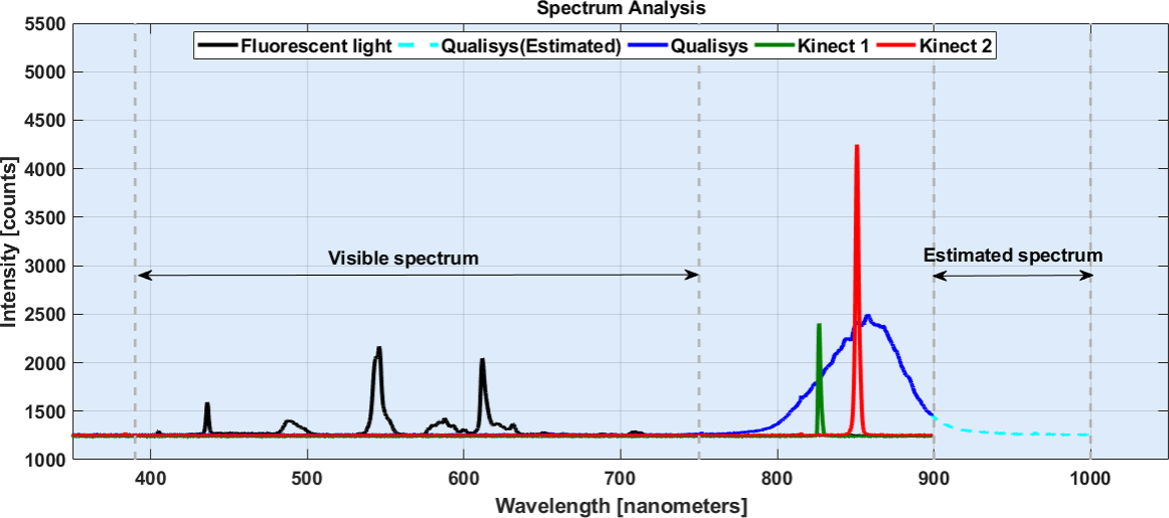
The spectrum of emitted lights from Kinect sensors and Qualisys motion capture system. The black curve shows the emitted light spectrum of fluorescent lights in the lab. While the spectrum of the projected IR ray from Microsoft Kinect v1 and Microsoft Kinect v2 are shown in green and red. The spectrum of emitted light from Qualisys Oqus 300/310 is shown in blue. However, a part of the curve with wavelength higher than 900nm were estimated.

In this section, a prior investigation on the spectrum of projected lights from Kinect sensors, Qualisys motion capture system and laboratory environmental light have been conducted. The emitted lights were recorded using an Ocean Optics 2000 spectrometer (Ocean Optics, Largo, USA) for each projector individually. The spectrometer is equipped with silicon detector, which can quantize the light intensity from 200nm up to 900nm. Fig 2 depicts the recorded spectrum using the spectrometer. Since the spectrometer recording spectrum was limited up to 900nm, the right side of projected beams for Qualisys camera was estimated based on expected distribution.

The pre-analysis showed a part of emitted strobes from the Oqus 300/310 cameras were beyond the measuring range of spectrometer (see Fig 2). Hence, by assuming the emitted light was normally distributed, the spectrum above 900 nm wavelength has been estimated.

Based on the estimation 95% of the projected lights from Qualisys cameras (Oqus 300/310) were within range of 800nm to 900nm with the peak intensity in 854nm. Similarly, Microsoft Kinect v2 projectors emitted NIR rays with 850nm peak intensity but very narrow bandwidths (842.7nm to 859.23nm). Whereas Microsoft Kinect v1 emitted structured pattern within 812.55nm to 841.6nm spectrum, and peak intensity was 827nm (see Fig 2).

## Previous studies

Several studies have investigated the accuracy and precision of the estimated depth maps in both generation of the Microsoft Kinect sensors and provided noise models for the sensors [35,41,42]. Mallick et al. [43] divided Kinect noise sources into spatial noise, temporal noise, and interference noise.

Spatial noise might come from axial error, lateral error, object medium, and sensor specifications [43]. Nguyen et al. [44] modeled the axial and lateral noise in Kinect v1 using a flat surface with quadratic and linear functions, respectively. Choo et al. [37] improved the noise models using both flat surface and a 3-dimensional checkboard. In contrast, Pagliari and Pinto [45] showed that the axial noise level in Kinect v2 is more stable at different depths and that it is not a quadratic model compared to the Kinect v1. They modeled the error in the estimated depth in Microsoft Kinect v1 with a second-order polynomial function increases by the distance from the target object, while they reported Microsoft Kinect v2 introduces much less error which slightly increases linearly by distance. Fankhauser et al. [41] showed the axial noise is significantly increased by increasing angle of the Kinect v2 sensor with respect to the surface.

The object medium plays an essential role in estimating its depth. Previous studies found that Kinect sensors could not correctly evaluate the position of objects with any transparent, reflective, or IR light-absorbing materials such as water bottles, mirrors or leather fabrics [43,46].

In the literature, two sources of interference were presented: ambient light and multiple sensors. The evidence showed Kinect v1 to have poor performance in the presence of high intensity of wide-spectrum ambient light such as sunlight or halogen lamps [47,48], while, Kinect v2 is more robust to the same disturbing light sources [41,48].

Using multiple Kinect sensors in the same generation could also cause interference between Kinect sensors. The impact of the interference between two Kinect v1 sensors was evaluated in several studies, and the results indicated considerable distortion when the angle between the two sensors was less than 60 degrees [49–51]. Sarbolandi et al. [48] showed that using two Kinect v1 increased the unknown depth areas about 10% while using two Kinect v2 sensors simultaneously introduces repetitive interference between the sensors.

## Materials and methods

### Data collection

In this study, five series of recordings in the different distance was performed to evaluate mutual interference between the Microsoft Kinect v2 and a marker-based motion capture system as a golden standard for tracking physical activities. In this study, the Qualisys passive marker-based motion capture system had been utilized as a golden standard.

The region of interest (ROI) was provided by hanging a bulletin board in the middle of the laboratory. The size of the bulletin board was 120cm×120cm and covered by non-reflective, white coarse cotton fabric. Four retroreflective markers were placed on the corners of the bulletin board (by a margin of 5cm) to track the bulletin board position using the Qualisys Track Manager.

Eight Qualisys Oqus 300/310 cameras were pointed to the ROI. The recordings were carried out using a Qualisys Track Manager (QTM) 2.9 (build 1697) with the 250Hz sampling frequency and 200μs exposure time. A Microsoft Kinect v2 sensor was utilized to evaluate possible distortion on each Kinect sensor.

The Qualisys cameras were adjusted, where five of the cameras had the scene of the bulletin board in the middle of the recording area while the other three cameras were behind the bulletin board.

**Fig 3:**
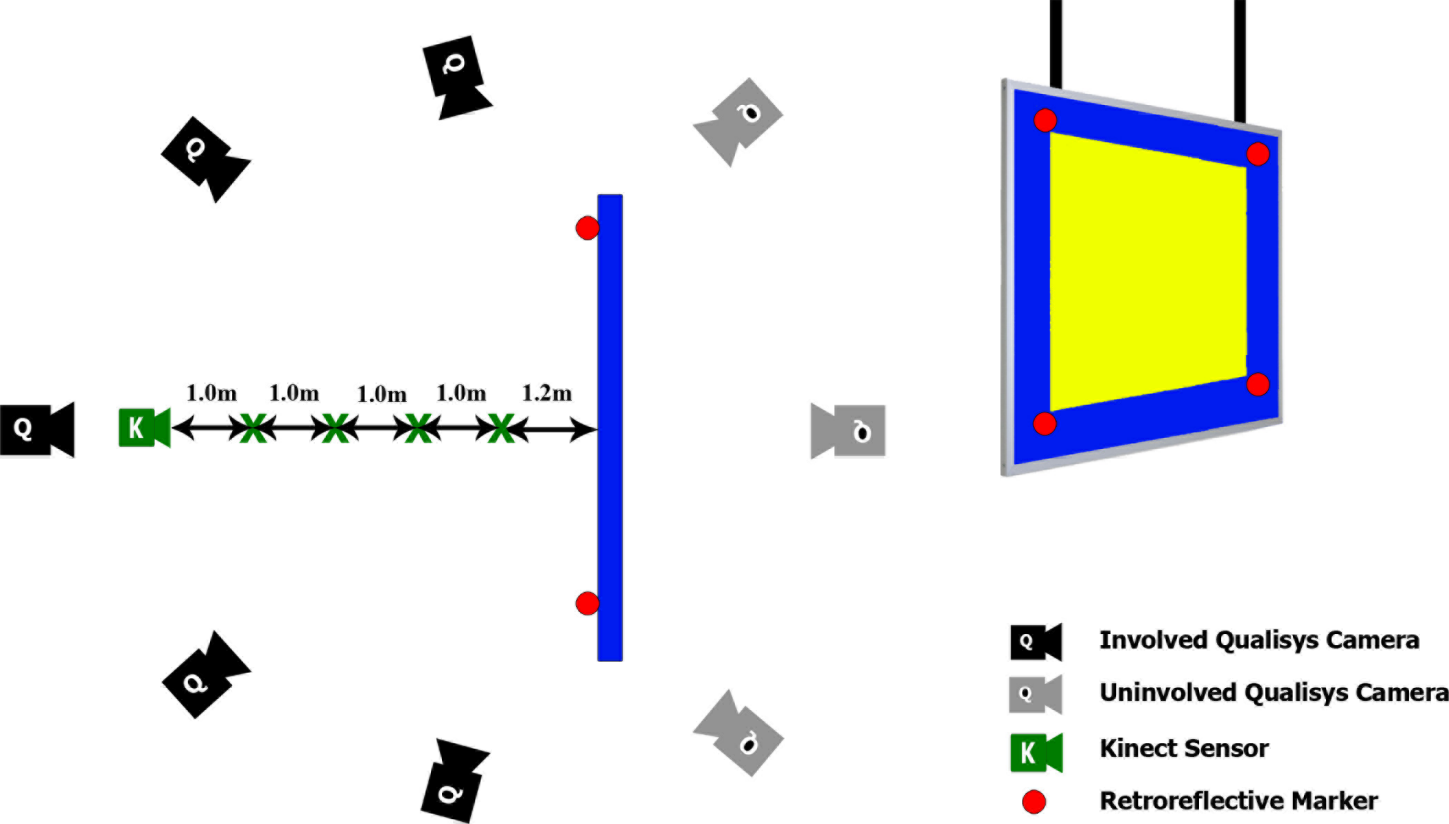
Geometric placement of sensors and bulletin board in the motion lab. Microsoft Kinect v2 was utilized, and 8 Qualisys cameras are mounted in the laboratory (top view). Only five cameras pointed to the bulletin board surface (i.e., involved cameras), and other cameras were placed behind the bulletin board (i.e., uninvolved cameras). The Kinect sensor was 1.2m of the board and moved up to 5.2m with 1.0m steps. The region of interest (ROI) on the bulletin board surface is shown with a yellow color.

The Microsoft Kinect v2 was placed 120cm from the bulletin board covering the whole surface of the bulletin board in the first series of recording. For the next four recording, the Kinect sensor moved away with steps of 100cm. The distance between the sensor and bulletin board was chosen within the recommended rage of working with the Microsoft Kinect v2 for capturing depth images [18,52]. The distance between the bulletin board and the sensor was measured using a Leica DISTO D2 (Leica, Wetzlar, Germany) laser distance meter. The sensor was pointing roughly perpendicular to the bulletin board surface while the center of RGB cameras was adjusted at the center of the bulletin board (see Fig 3).

A customized application was developed to capture and record depth from Microsoft Kinect using Microsoft Kinect SDK version 2.0. The application was developed using Visual Studio 2015, update 3, under Windows Presentation Foundation (WPF) Application Programming Interface (API). Depth information was stored in lossless 16bit PNG image compression with 30 frames per seconds, and for each distance, the depth images were captured for 10 minutes.

To establish global timing in the setup, a server application was developed to communicate with the Microsoft Kinect recording apps and Qualisys Track Manager using Qualisys Track Manager Real-Time (QTM-RT) protocol v1.12. The simultaneous recording ensured using TCP/IP command control through the network, and the between recorder latency kept below 4ms.

Since a small temperature drift in the Kinect sensor has been reported [48], Kinect v2 was turned on 30 minutes prior to the recordings. In addition, the ambient room temperature was controlled (25°c room temperature) while motion lab light was provided by fluorescent light only.

### Test protocol

In this study, the impact of the motion capture system on the Microsoft Kinect v2 depth recordings was investigated by using a flat surface (ROI). Depth information of the ROI was captured and stored using a Microsoft Kinect v2 sensor.

To examine the mutual interference, we assumed the recordings referred to a flat surface in the space (i.e., the bulletin board surface). Therefore, the following assumptions were considered, and the employed approach should satisfy them.

**Fig 4:**
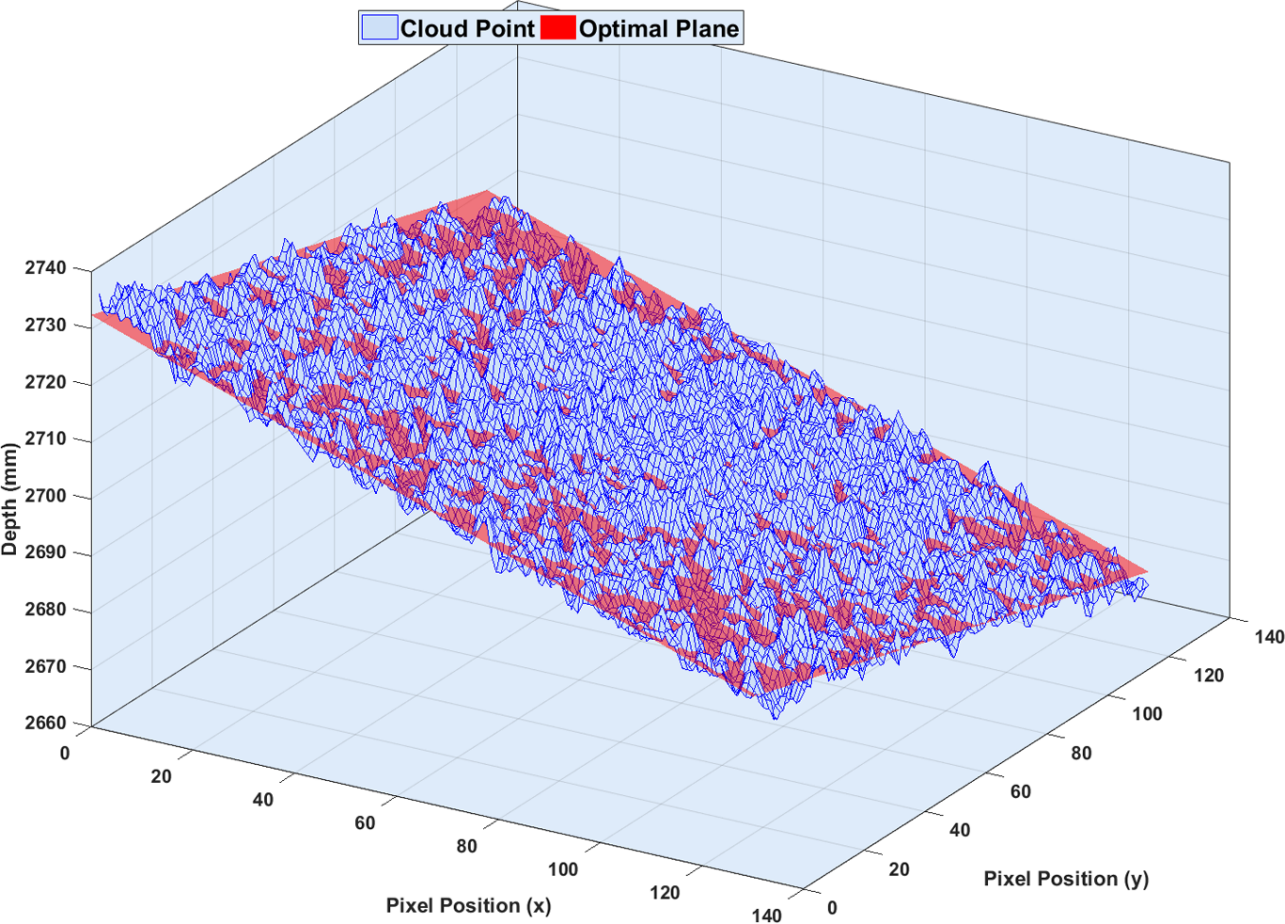
Defining an optimal plane. Based on a single depth map, the optimal plane was determined based on the cloud point while trying to minimize the error rate. The blue mesh represents the cloud points of the bulletin board captured by Microsoft Kinect v2 (depth information), and the corresponding optimal plane is shown in red.

1. The smoothness of the bulletin board surface was stable during the recording (the bulletin has a rigid surface);
2. The board might have swung slowly, and the swing rhythm was unpredictable. Therefore, the calculation should be independent to movements of the bulletin board.
3. The accuracy of the Qualisys motion capture system did not change during the experiments;

Consequently, we assumed an optimal plane that satisfied cloud point on the bulletin board surface. The optimal plane was estimated by calculating the average position of point cloud *P*^*n*×3^, and assuming the average position satisfied the plane equation. Therefore, the normal vector of the optimal plane was calculated based on (1).

$$N^{1\times 3}=eigen\left({\left(P^{n\times 3}-M^{1\times 3}\right)}^{T}\times (P^{n\times 3}-M^{1\times 3})\right)$$ (1)

where *N*^1×3^ is the normal vector of the optimal plane and M is the average position of the cloud point. In this equation, the cloud point in the Kinect records was the depth maps, and similarly, an optimal plane can be introduced by the reflective marker positions in the Qualisys records. Fig 4 represents an optimal plane based on a single Kinect v2 point cloud.

As can be seen in Fig 4, the acquired bulletin board had some degree of roughness, which might be due to the real roughness of the bulletin board surface or to the resolution of the Kinect. Hence, the residual value for each depth frame (i) in each pixel position (x,y) was defined by calculating Euclidean distance between recorded depth and estimated depth based on the optimal plane.

$${Residual}_{{Frame}_{i}}(x,y)=\|D_{i}(x,y)-D_{i}^{ref}(x,y)\|$$ (2)

In this equation, *D*_*i*_(*x*,*y*) represents recorded depth in x and y position, while $D_{i}^{ref}(x,y)$ stands as the expected depth from the optimal plane in the record frame i. Therefore, the roughness of the region of interest has been estimated by calculating root mean squares (RMS) of residual values in each frame. Hence, for each frame, residual root mean squares (RRMS_Frame_) were calculated according to (3).

$${RRMS}_{Frame}(i)=\sqrt{\frac{1}{m.n}\sum_{x=x_{0}}^{n}\sum_{y=y_{0}}^{m}{\left(D_{i}(x,y)-D_{i}^{ref}(x,y)\right)}^{2}}$$ (3)

where, m, n, *x*_0_ and *y*_0_ limit the calculations to the area on interest. The surface roughness was assessed along with an estimate of entropy; these estimates were used to measure the impact of the noise sources on the region of interest. The roughness of each pixel position was also involved in estimations instead of calculating the roughness of each frame. Accordingly, the residual root mean square value of RRMS_Pixel_ was calculated based on (4).

$${RRMS}_{Pixel}(x,y)=\sqrt{\frac{1}{n}\sum_{l=1}^{n}{\left(D_{i}(x,y)-{D_{i}}^{ref}(x,y)\right)}^{2}}$$ (4)

Where n is the number of images in each record and x and y stand as the position of the pixel in the region of interest. Entropy was estimated using (5), where *p*_*x*,*y*_ is the probability of observed value in the pixel_x,y_.

$$Entropy(x,y)=-\sum p_{x,y}{log}_{2}(p_{x,y})$$ (5)

In this study, for simplifying the possible noise sources, the interferences can be divided into passive and active distortion. As a result, the near-infrared laser projector of Kinect sensor and the projector of each camera in the motion capture system are considered as active sources of distortion, and the retroreflective markers in this study were categorized as a passive noise source.

## Result

### Residual root mean squares (RRMS)

The statistical analysis of the RRMS_frame_ values indicated the calculated values were not normally distributed (Shapiro-Wilk normality test p< 0.05). Consequently, median and interquartile range (IQR) of calculated RRMS_Frame_ in absence and presence of Qualisys system as noise source are shown in Table 2. In addition, a statistical test of each paired recording was investigated by two-sided Wilcoxon test.

**Table 2:** Median and IQR of estimated RRMS_frame_ using Microsoft Kinect v2 in absence and presence of the motion capture as a noise source for five different distance of the Kinect sensor from the board.

Distance from the bulletin board (cm)	Median and IQR of RRMS_frame_ (mm)		p-value
	In the absence of Qualisys	In the presence of Qualisys	
120 cm	1.45±0.02mm	1.60±0.15mm	<0.001
220 cm	1.61±0.02mm	2.00±0.22mm	<0.001
320 cm	2.54±0.05mm	3.56±0.88mm	<0.001
420 cm	3.47±0.09mm	4.89±1.06mm	<0.001
520 cm	4.53±0.13mm	6.84±1.46mm	<0.001

**Fig 5:**
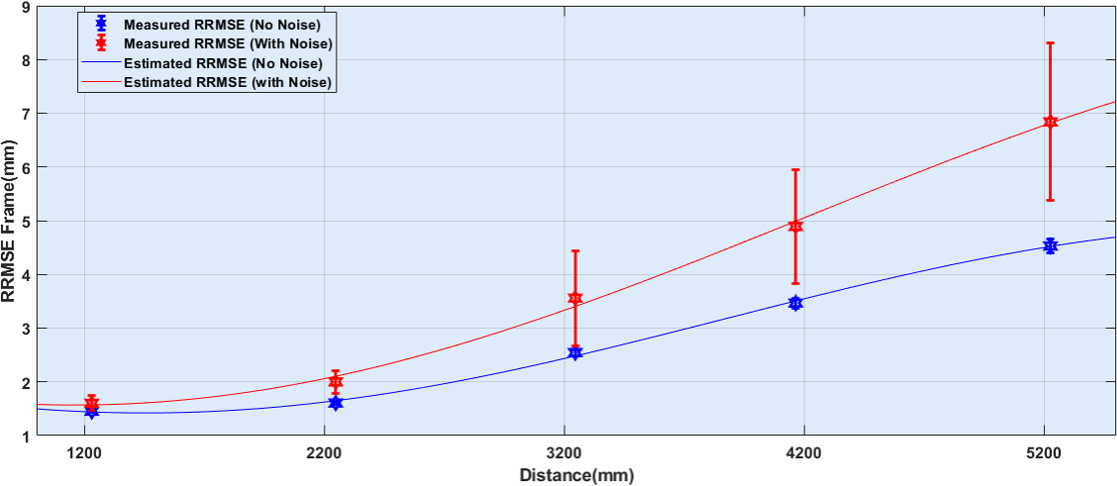
Measured RRMS_Frame_ values in absence and presence of Qualisys as an interference source in Kinect v2 depth records. The values represented the median of RRMS_Frame_ and range stand as corresponding IQR. The line graphs were interpolated based on the measurements.

The corresponding estimation of measured RRMS_Frame_ is worked out using the first order Fourier estimator, and it is shown in Fig 5.

The Bland-Altman analysis is employed to evaluate the impact of the motion capture system on the estimated RRMS_Frame_ by comparing the measurements in presence and absence Qualisys. The Bland-Altman analysis and corresponding limits of agreement and the bias of RRMS_Frame_ for each configuration were calculated. Bland-Altman analysis revealed bias and limits of agreements had an increasing trend by increasing distance to the surface as is shown in Table 3.

**Table 3:** Bland-Altman analysis of the estimated RRMS_frame_ using Microsoft Kinect v2 evaluating the impact of the motion capture as a noise source for five different distance of the Kinect sensor from the board.

Distance	Bias	95% LoA
120 cm	10.18mm	20.16
220 cm	24.58mm	26.33
320 cm	39.99mm	65.95
420 cm	40.88mm	58.50
520 cm	49.92mm	61.83

**Fig 6:**
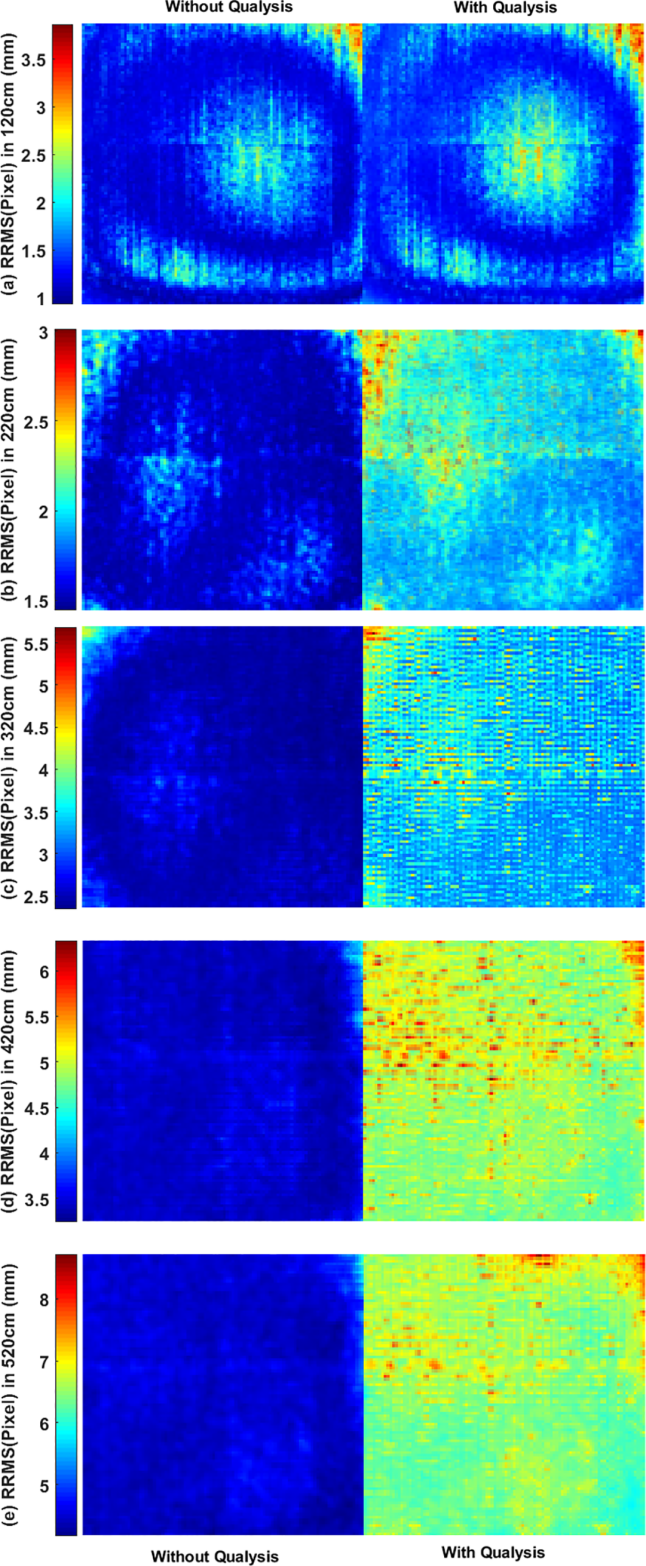
Comparing the impact of Qualisys on the estimated RRMS pixel in Microsoft Kinect depth records. Where Kinect was placed at (a) 120cm, (b) 220cm, (c) 320cm, (d) 420cm, (e) 520cm of the bulletin board (ROI). ROI dimension where 270×270, 150×150, 100×100, 80×80, 70×70 pixels from 1.2m to 5.2m. In the figure, all ROI images are resized to provide a better presentation.

Accordingly, RRMS_Pixel_ has been calculated based on (4) for each pixel in Kinect depth records. Fig 6 shows Qualisys increased RRMS_Pixel_ in Kinect v2 records. Apparently, the size of the ROI (in terms of the number of pixels) decreased by increasing distance from the bulletin board.

### Entropy

**Fig 7:**
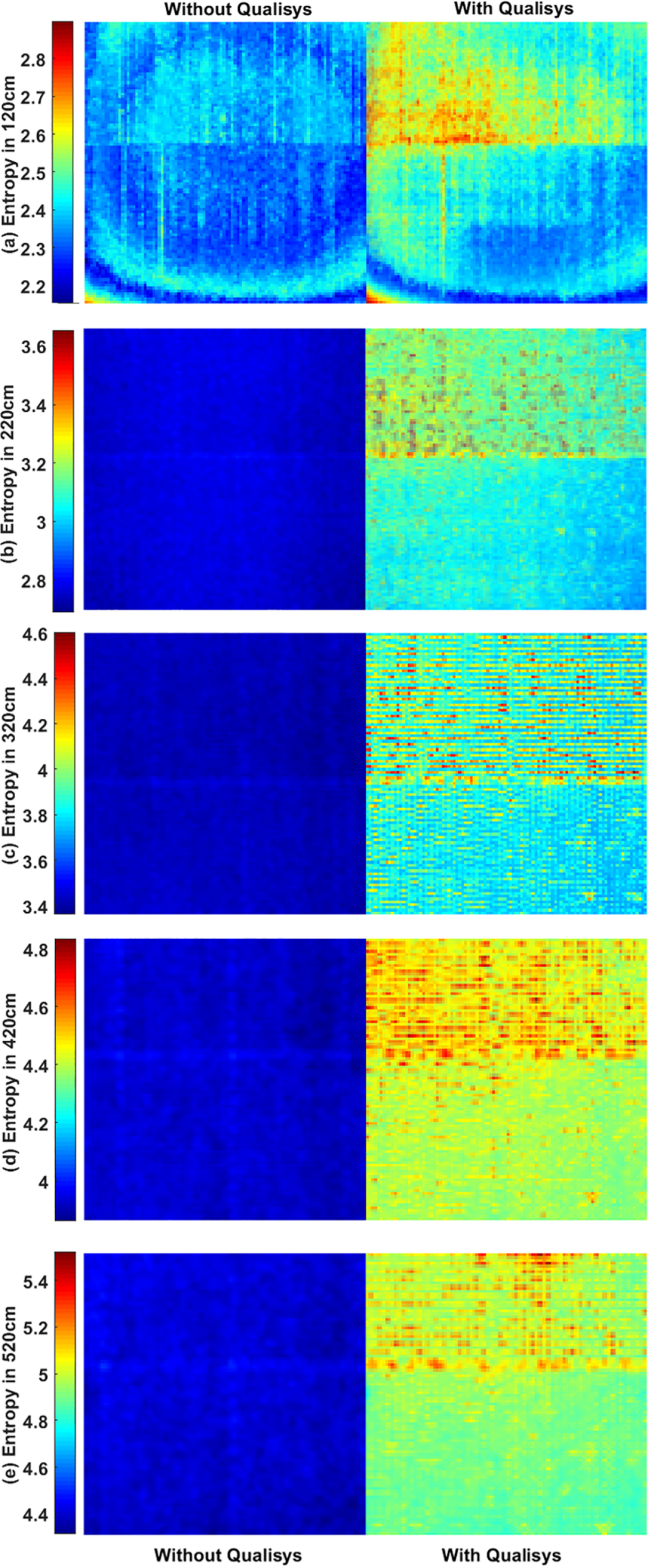
Comparing the impact of Qualisys on the entropy of Microsoft Kinect depth records. Where Kinect was placed at (a) 120cm, (b) 220cm, (c) 320cm, (d) 420cm, (e) 520cm of the bulletin board (ROI). ROI dimension where 270×270, 150×150, 100×100, 80×80, 70×70 pixels from 1.2m to 5.2m. In the figure, all ROI images are resized to provide a better presentation.

The pixel-wise entropy of recorded depth images was estimated based on (5). Fig 7 compares pixel-wise entropy with Kinect v2 depth records in absence and presence of Qualisys as a noise source in 5 different distances.

### Retroreflective markers and LED strobes

**Fig 8:**
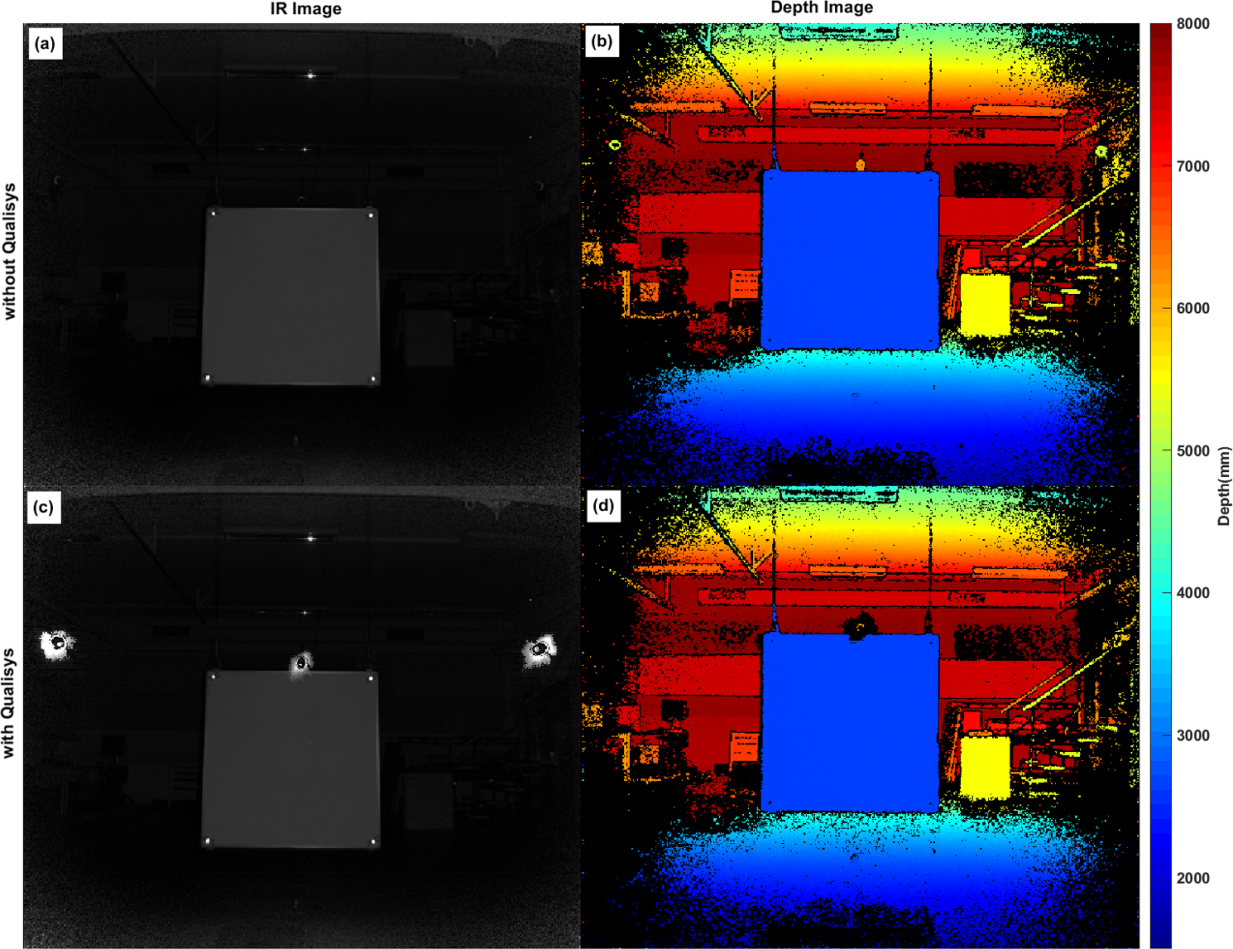
Impact of reflective markers and IR beams in Kinect v2. (a) an IR record of Microsoft Kinect v2 while Qualisys Oqus cameras were turned off and (b) corresponding depth information. (c) An IR record of Microsoft v2 while Qualisys Oqus cameras were turned on and (d) the corresponding depth information. The black areas in the depth images are representing the areas of unknown distance.

**Fig 9:**
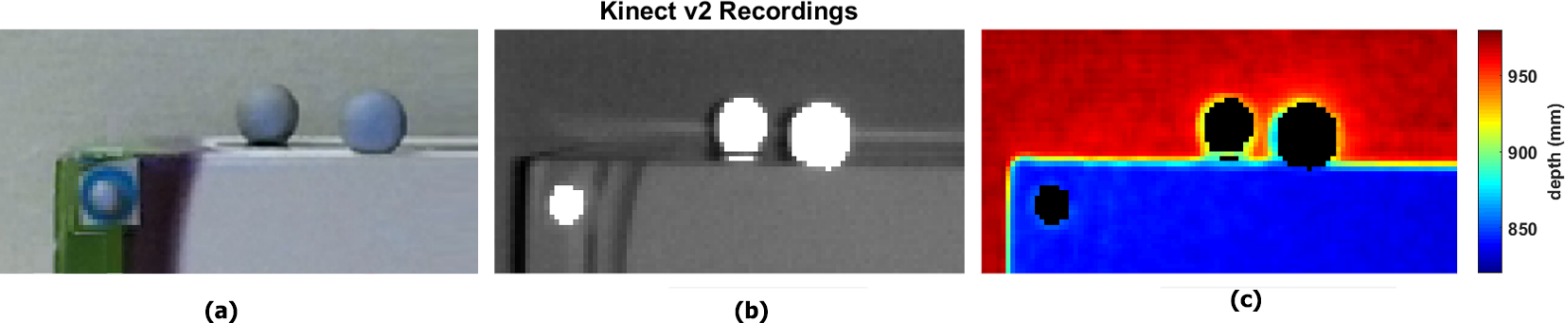
Impact of reflective markers on Microsoft Kinect v2 recordings. (a) RGB image, (b) IR image and (c) depth information. The black areas in the depth images are representing the areas of unknown distance.

The result showed Kinect v2 was also sensitive to the reflected light form retroreflective markers and Qualisys cameras (see Figs 1A, 8 and 9). Figs 8 and 9 show the impact of reflective markers and Qualisys cameras on IR and depth images. As can be seen in figures, not only the reflective markers that appear as bright spots but also the Qualisys cameras surrounded by an aureole of bright dots. Surprisingly in the depth image, reflective markers are seen like black holes of unknown distance. However, the reflective balls were not a part of ROI (Fig 3); the impact of reflective markers was investigated by assessing the heterogeneity of pixel on each corner. The assessment did not represent any significant changes due to the presence of reflective markers nearby the ROI.

## Discussion

This study evaluated the impact of a marker-based motion capture system on the Microsoft Kinect v2 sensor in five different distances. The interference was estimated based on captured raw depth images using Microsoft Kinect v2. It was hypnotized that by placing the bulletin board and Qualisys cameras in a steady position, the amount of the projected ray to bulletin board surface will be constant. Accordingly, the contribution of the motion capture system in all the measured distances as noise source will be the same.

The results showed that Kinect v2 recordings are sensitive to the presence of the Qualisys cameras and that they might have a destructive impact on Kinect v2 measurements and post-processing calculations. Consequently, certain degrees of uncertainty might be imposed on Kinect v2 measurement. To the best of our knowledge, none of the previous studies have reported Kinect v2 depth information distortion due to a motion capture system, and retroreflective markers.

In Fig 5, it can be seen by increasing distance from the ROI sensitivity of the Microsoft Kinect is rising and the distortion is nonlinear toward the distance.

The pixel-wise RRMS and entropy also indicate the motion capture system has a considerable impact on the Microsoft Kinect recordings and it increases by increasing the distance (see Figs 6 and 7).

Having examined Figs 6 and 7, it can clearly be seen in the lens distortion on the depth recording when the Kinect sensor was placed at 120cm of the ROI. By increasing the distance, the lens distortion disappeared gradually.

Hence, it may be concluded the Qualisys motion capture system has a clear impact on the Kinect v2 depth estimation. This interference distorts the depth maps continuously and with a high variation.

Retroreflective markers were also a source of passive noise as depicted in Fig 9. Thus, retroreflective markers in the Kinect v2 IR images appeared like bright balls, resulting in areas of unknown distance (depth) in the depth map. The figure reveals not only areas of unknown distance in the marker position, but also wrong distance values surrounding the silhouettes of the marker edges.

It can be concluded that two factors are involved in a motion capture system interfering with Microsoft Kinect recordings. First, the emitted light from the motion capture system should be within the same range of Microsoft Kinect sensor sensitivity. Secondly, the intensity of distortion on Kinect measurements might be highly dependent on the depth images reconstruction approach.

Fig 2 indicates Qualisys cameras project a wide spectrum NIR lights (from 800nm to beyond 900nm) than Kinect v2, while Kinect v1 and Kinect v2 emit narrowband NIR lights centered at 825nm and 850nm respectively. Therefore, we may conclude Kinect sensor is sensitive to projected light from Qualisys cameras. This satisfies the requirement for interfering with Kinect recordings.

Regarding the result, we can conclude, ToF based depth sensors (Kinect v2) are potentially sensitive to the motion capture system. Whereas, Qualisys utilize a fast time division between mounted LED strobes on each camera. Consequently, it might be the primary cause of interference with the intensity modulation in Kinect v2.

This study had three limitations. First, only depth data of Microsoft Kinect v2 were analyzed to investigate the contribution of possible noise on the recordings. However, Microsoft Kinect skeleton algorithm might also be affected by the motion capture system. In addition, only a steady and flat surface was chosen to evaluate the noise impact on Microsoft Kinect v2 depth data recordings. Finally, in this study due to the lack of detail information about Microsoft Kinect v2 working principle, we were not able to provide a model for the shown noise in the presence of the motion capture system.

In the current study, only the impact of the Qualisys motion capture system (as a marker-based motion capture system) on Kinect sensor recordings was assessed. Other motion capture systems might have a different destructive impact. In theory, all the marker-based motion capture systems might interfere with Microsoft Kinect v2 recordings. Therefore, it is recommended that Kinect v2 sensitivity to the presence of motion capture systems should be investigated before both systems are used together. It could be hypothesized that the actual validity and reliability of Microsoft Kinect v2 (depth data and accordingly estimated skeleton) might be higher the reported values in the previous studies due to the active and passive noises from the motion capture systems.

## Conclusions

The influence of passive and active noise sources on depth assessments by Microsoft Kinect sensor and Qualisys motion capture system were evaluated. The findings indicate that the Kinect v2 sensor is not only affected by the Qualisys motion capture system but also that the presence of retroreflective markers plays an essential role in producing misleading Kinect v2 measurements.
